# Plasma cell-free tumor DNA, *PIK3CA* and *TP53* mutations predicted inferior endocrine-based treatment outcome in endocrine receptor-positive metastatic breast cancer

**DOI:** 10.1007/s10549-023-06967-3

**Published:** 2023-06-21

**Authors:** Tom Wei-Wu Chen, Wen Hsiao, Ming-Shen Dai, Ching-Hung Lin, Dwan-Ying Chang, I-Chun Chen, Ming-Yang Wang, Shu-Han Chang, Shu-Min Huang, Ann-Lii Cheng, Ko-Wen Wu, Kien Thiam Tan, Yen-Shen Lu

**Affiliations:** 1 Department of Oncology, National Taiwan University Hospital, Taipei, Taiwan; 2 Graduate Institute of Oncology, National Taiwan University College of Medicine, Taipei, Taiwan; 3 Department of Medical Oncology, National Taiwan University Cancer Center, Taipei, Taiwan; 4 ACT Genomics Co., Ltd, Taipei, Taiwan; 5 Division of Hematology and Oncology, Department of Medicine, Tri-Service General Hospital, Taipei, Taiwan; 6 Department of Surgery, National Taiwan University Cancer Center, Taipei, Taiwan

**Keywords:** Liquid biopsy, Cell-free tumor DNA, Breast cancer, Endocrine receptor

## Abstract

**Purpose:**

How to factor both tumor burden and oncogenic genomic mutations as variables to predict the outcome of endocrine-based therapy (ET) in ER-positive/HER2-negative metastatic breast cancer patients (MBC) remains to be explored.

**Method:**

Blood samples prospectively collected from 163 ER-positive/HER2-negative female MBC patients, before ET, were used for cell-free tumor DNA (cfDNA) analysis. cfDNA was subjected to next-generation sequencing (NGS) to interrogate oncogenic *PIK3CA* hotspot and *TP53* DNA-binding domain (DBD) mutations, including single nucleotide variants (SNVs) or small insertions and deletions (InDels). The variant calling threshold was set at 0.5%. Progression-free survival (PFS) was measured from the start of the ET treatment to the time of disease progression of the same treatment regimen.

**Results:**

Overall, the median PFS was 8.3 months (95% CI 5.7–11.1 months). The median cfDNA was 38.5 ng (range 4.4–1935 ng). The proportion of patients with *PIK3CA* and *TP53* alterations were 25.1 and 15.3%, respectively. Patients with high total cfDNA (HR 1.74, *p* = 0.003), *PIK3CA* mutation (HR 1.74, *p* = 0.007), and *TP53* mutation (HR 1.64, *p* = 0.047) in liquid biopsy conferred worse outcome after ET. Even for patients with low tumor burden, the detrimental effect of *PIK3CA* or *TP53* mutation remained significant (*p* < 0.001). For patients with either *PIK3CA* (*p* < 0.001) or *TP53* mutation (*p* = 0.004), there was significant positive correlation between allele frequency (AF) and total cfDNA.

**Conclusion:**

After adjustment of cfDNA level, *PIK3CA* and *TP53* mutations observed in liquid biopsy exerted detrimental effects on the outcome of ET-based regimens. The AF of *PIK3CA* or *TP53* may be a surrogate marker for PFS.

**Supplementary Information:**

The online version contains supplementary material available at 10.1007/s10549-023-06967-3.

## Introduction

Despite the progress in the endocrine therapy (ET)-based regimens of endocrine receptor (ER)-positive human epidermal growth factor receptor (HER2)-negative metastatic breast cancer (MBC), the prognosis and the survival outcome of different treatment regimens vary far and wide. A better estimate of treatment outcomes could facilitate communication of physicians and patients and the selection of the optimal treatment for these patients.

Tumor burden plays a critical impact on prognosis but it may not be easily quantified and estimated for each patient. For ER-positive MBC patients, estimating tumor burden may be further complicated by the common involvement of bone metastases that is difficult to measure the extent and size of metastatic tumors. The genomic DNA of cancer cells could shed into the bloodstream in the form of cell-free tumor DNA (cfDNA) and is characterized by shorter fragmented reads, ranging from 100 to 200 base pairs [1]. Studies in many cancer types have shown that the total amount of cfDNA is a reflection of total tumor burden and associated with prognosis [1–6]. In addition, these cfDNA could also be used as sources for identification of of specific genetic alterations that are also important in the evaluation of prognosis [7–9].

*PIK3CA* and *TP53* are two genes that have been shown to affect the treatment outcome and prognosis of MBC. As *PIK3CA* activation via mutation is one the resistance mechanisms of chemotherapy and anti-HER2 therapy [10, 11], some studies have shown that a ET-based combination regimen may still provide similar proportion of benefit to both *PIK3CA* wild type and mutant patients [12, 13]. However, because these studies may incorporate patients with a wide range of tumor burden, parsing out the factor of tumor burden may provide insights to our understanding of the clinical impact of *PIK3CA* mutation. The tumor suppressor *TP53* is one of the most common mutated gene in cancer but it’s role in MBC is less investigated. Although *TP53* alterations are more common in triple-negative breast cancer [6], *TP53* had strong negative prognostic impact when present in ER-positive early breast cancer [14]. Despite studies have suggested that alterations of oncogenic mutations *TP53* are associated with worse outcome [15], few incorporated or adjusted the factor of tumor burden in their studies.

Liquid biopsy of a patient’s blood could provide information regarding the total cfDNA as well as tumor-specific genomic alterations via next-generation sequencing (NGS) [1, 2]. Albeit there are multiple research- or commercial-based cfDNA targeted panel of liquid biopsy in the clinical field, few integrated tumor burden and oncogenic mutations of breast cancers into the profiling and its association with treatment outcome.

In this study, we aim to prospectively collect ET-based treatment outcome of ER-positive HER2-negative MBC patients and the clinical utility of liquid biopsy cfDNA testing. Instead of collecting a broad mutation profile, we designed a targeted amplicon sequencing panel focused on oncogenic mutations of the two most common mutations in ER-positive breast cancer, namely *PIK3CA* and *TP53* and incorporated the total cfDNA as one of the reporting endpoints. Our study provides evidence that a focused panel of genes and inclusion of total cfDNA is adequate to provide insights of the impact on ET-based treatment for ER-positive HER2-negative MBC patients.

## Patients and methods

### Patient enrollment

ER + /HER2- MBC patients were eligible if they were older than age 20 and were intended for ET-based regimens. ET included single agent endocrine therapy agent or in combination with targeted agents such as cycline-dependent kinase (CDK) 4/6 inhibitor or everolimus (a mammalian target of rapamycin (mTOR) inhibitor). Patients who received endocrine therapy with metronomic chemotherapy were also eligible. The determination of ER and HER2 status was according to the ASCO guideline as previously reported [16, 17]. Repeated biopsy after metastatic diagnosis was not required and the status of ER and HER2 was based on the most recent pathological report (primary or metastatic pathological reports were both eligible). Subgroup analysis for the ET plus everolimus subgroup was pre-specified in the protocol. This study was approved by the Research Ethics Committee of National Taiwan University Hospital (201411008RIND & 201703138RIPD) and Institutional Review Board of Tri-Service General Hospital (1-107-05-003).

### DNA extraction and NGS

Blood sample was collected in the Cell-Free DNA Blood Collection Tubes (BCT, Streck, La Vista, NE) [18] followed by a two-step centrifugation (1600×*g* then 17000×*g*, both for 10 min at room temperature) separation procedure. After separation, 2.5–4 ml of plasma was used to extract cfDNA with the QIAamp circulating nucleic acid kit according to the manufacturer’s protocol (Qiagen). DNA concentration and integrity were determined using the Quant-iT dsDNA HS Assay (Invitrogen) and the Fragment Analyzer (Advanced Analytical Technologies, Inc.), respectively. A total of 20 ng of cfDNA would be was subsequently PCR-amplified for 26 cycles with a multiplex panel consisting of primer pairs covering regions of *TP53* DNA-binding domain (DBD) and hotspot *PIK3CA* mutations, which included a total of 13 amplicons containing 1137 base pairs. PCR products were ligated to barcode adapters and underwent further amplification, followed by emulsion PCR on the OneTouch System (Applied Biosystems). A maximum of 96 samples were pooled on the Ion PI™ Chip Kit on Ion Proton System with the aim for an average of 10,000 × average read depth. Twelve healthy volunteer’s PBMC were used as control.

### Variant analysis

The analyzed variants included single nucleotide variants and small insertions and deletions with the limitation of variant detection set at 0.5%. The human genome sequence hg19 was used as the reference genome, and alignment and base calling were performed with the Torrent Suite Server version 4.4. Annotated plasma variants had to have at least 20 reads and the allelic fraction had to be above a background threshold of 7 Z-Scores from the mean of healthy donors. When developing this NGS assay and analysis, we assumed each sequencing position will be covered 1000 × reads. Due to the limit of detection being set to 0.5%, the variant read counts need to be larger than 20 read counts for further manual check. Annotated tumor variants had to have a variant frequency of at least 0.5%, or to be known hotspot variants identified as true variants after raw data analysis. For annotation, COSMIC (v74), dbSNP 138 and 1000 Genomes of the global population data (phase1) were used.

### Statistical analysis

Progression-free survival was defined as the date of start of the treatment to the date of evidence of disease progression. Log-rank test and Cox proportional hazard methods were applied for the testing of genomic alteration and PFS outcome. Linear regression and Pearson correlation coefficients were used to examine the correlation between the allele frequency (AF) of cfDNA genomic alterations and PFS outcome. All statistical tests were performed using R 3.6.3.

## Results

From Aug 2015 to May 2020, a total of 163 ER-positive HER2-negative MBC patients were prospectively enrolled into the study. All patients were women. The median age was 60 (range 32–92). The lines of treatment that patients intended to receive following the enrollment was 20, 34, 31, 15% for first-line, second-line, third-line, and fourth or later-lines, respectively. All patients received at least an endocrine therapeutic agent as part of the regimen. Thirteen percent of patients received ET as single agent, 48% received ET plus everolimus, 14% received ET plus a CDK4/6 inhibitor, and 17% of patients received ET plus metronomic chemotherapy. Fourteen (8.5%) patients received fulvestrant as part of the ET regimen. The detail treatment regimens are listed in Table 1.

**Table 1:** The endocrine-based treatment regimens and the distribution of lines of the treatment in our study population and the progression-free survival time associated with each clinical group

Line of treatment	Patient number (%)	Median PFS months (95% CI)
1st	32 (20)	23.0 (5.3–NR)
2nd	56 (34)	11.8 (6.6–15.6)
3rd	50 (31)	5.8 (4.7–9.8)
4th and later	25 (15)	8.8 (4.1–14.2)
*Treatment regimens*		
ET only	21 (13)	15.6 (5.4–NR)
ET + CDK4/6 inhibitor	25 (15)	NR (8.4–NR)
ET + everolimus	78 (48)	5.8 (4.7–9.3)
ET + metronomic chemotherapy	27 (14)	7.8 (4.4–15.2)

*CDK* cyclin-dependent kinase, *CI* confidence interval, *ET* endocrine therapy, *NR* not reached, *PFS* progression-free survival

### The mutation profiles in ER-positive HER2-negative MBC patients

Using a 0.5% cut-off threshold for variant calling (see Patients and Methods), 57 (34.9%) patients had at least one *PIK3CA* or *TP53* mutations detected in the plasma with 41 (25.1%) and 25 (15.3%) patients harbored *PIK3CA* and *TP53* mutations, respectively. The proportions of patients with at least one genomic alteration did not differ significantly between lines of ET treatment (*p* = 0.81 for *PIK3CA*, *p* = 0.42 for *TP53*).

**Fig. 1:**
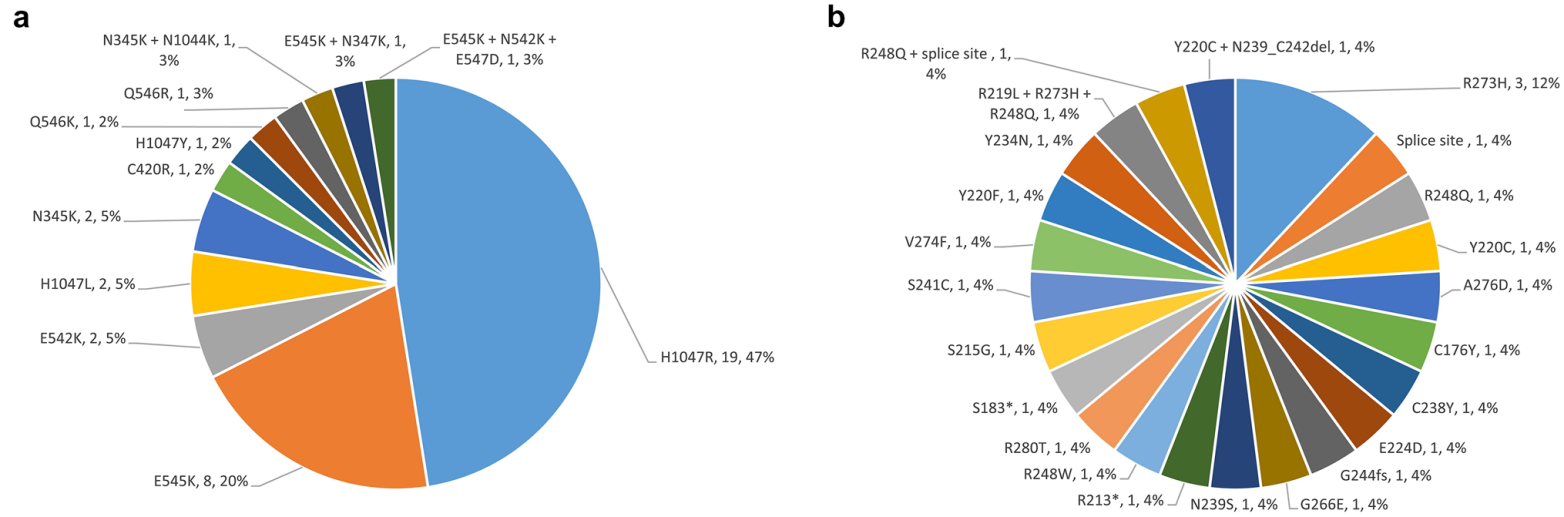
The mutation and indel profiles of **a** PIK3CA **b** TP53. The number after the specific genomic alteration indicates the number of patients

All patients with detectable *PIK3CA* cfDNA had single-point mutation and no small indels were detected. Three (8%) patients had 2 different *PIK3CA* mutation genotypes detected by liquid biopsy whereas all other patients had only one specific PIKCA mutation. The most common PI3KCA mutation hotspot was found at H1047 (53% of all *PIK3CA* alterations, including H1047R, H1047L and H1047Y). All identified *PIK3CA* mutations by liquid biopsy are shown in Fig. 1a. As for *TP53*, point mutations, frameshift changes and deletions (Fig. 1b) were identified. Owing to amplicon design limitations, all cfDNA *TP53* mutations were located within the DNA-binding domain.

### The effects of tumor burden on treatment outcome

Inclusive of all patients, the median PFS was 8.3 months (95% CI 5.7–11.1 months). The duration of PFS of each individual ET-based regimen and each line of ET-based regimens are shown in Table 1.

**Fig. 2:**
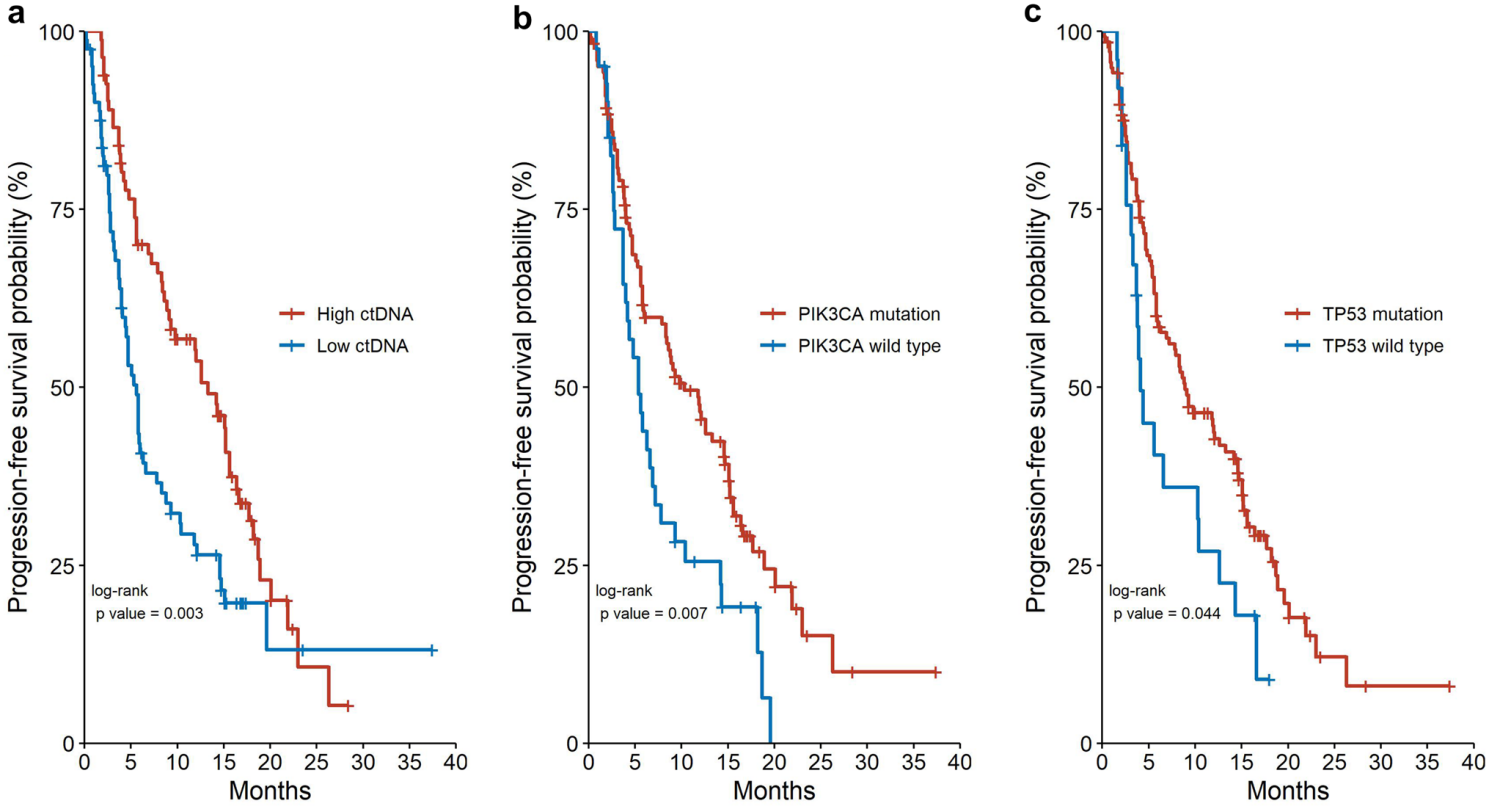
The Kaplan–Meier progression-free survival curves of **a** ctDNA (high vs low), **b** PIK3CA (mutation vs wild type), **c** TP53 (mutation vs wild type)

In here we relate tumor burden not only to the sheer volume of the disease but also to the aggressiveness of the tumor, as in contrast to some patients may have a moderate to high volume of tumor mass but with an indolent disease pattern. As cfDNA in the circulation is largely made up by genomic DNA fragments released from apoptotic or necrotic cancerous cells as a result of rapidly proliferation, the amount of cfDNA recovered can be used as surrogate for tumor burden evaluation [19, 20]. We also applied a cfDNA-tailored blood collection tube and algorithm to minimize the bias from normal cell circulating DNA [18]. We then explored the effect of tumor burden, as represented by total cfDNA, and oncogenic mutations *PIK3CA* and *TP53* on the outcome of ET-based regimens. The median amount of recovered cfDNA in the cohort was 38.5 ng (range 4.4–1935 ng) (The total cfDNA histogram is shown in Supplementary Fig. 1). Using median of 38.5 as the threshold for high tumor burden, we found that patients with higher tumor burden was significantly associated with a worse outcome after ET-based regimens (median PFS 5.6 vs 13.3 months, log-rank *p* = 0.003, HR 1.74, 95% CI 1.12–2.51,) (Fig. 2a).

### The effects of PIK3CA and TP53 oncogenic mutations on treatment outcome

Patients with *PIK3CA* mutation had worse PFS as compared to those without (median PFS 4.5 vs 10.3 month, log-rank *p* = 0.007, HR 1.74, 95% CI 1.16–2.62, Fig. 2b). We also compared the PFS results between patients with kinase domain (H1047X) mutation and other non-kinase domain *PIK3CA* mutations and found no significant differences (H1047X vs others median PFS 4.8 vs 5.8 months, *p* = 0.9). Moreover, plasma *TP53* mutations also exerted an inferior outcome (median PFS 4.1 vs 8.9, HR 1.64, 95% CI 1.01–2.67, *p* = 0.044, Fig. 2c). Changes of *TP53* protein were classified as missense or truncated based on predicted amino acid changes of the mutations and we did not find significant differences between the PFS results of these two groups (missense vs truncated *TP53* 5.6 vs 4.0 months, *p* = 0.23). The detrimental impact of *TP53* on PFS remained significant in *PIK3CA* wild type patients (HR 3.28, 95% CI 1.44–7.48, p = 0.0048). We also examined the differences in median PFS between double WT, PIK3CA mutant only, TP53 mutant only, and patients with both PIK3CA and TP53 mutations. The PFS between the three groups with any mutations were not significantly different (Supplementary Fig. 2).

### Correlation between tumor burden and PIK3CA/TP53 mutations

**Fig. 3:**
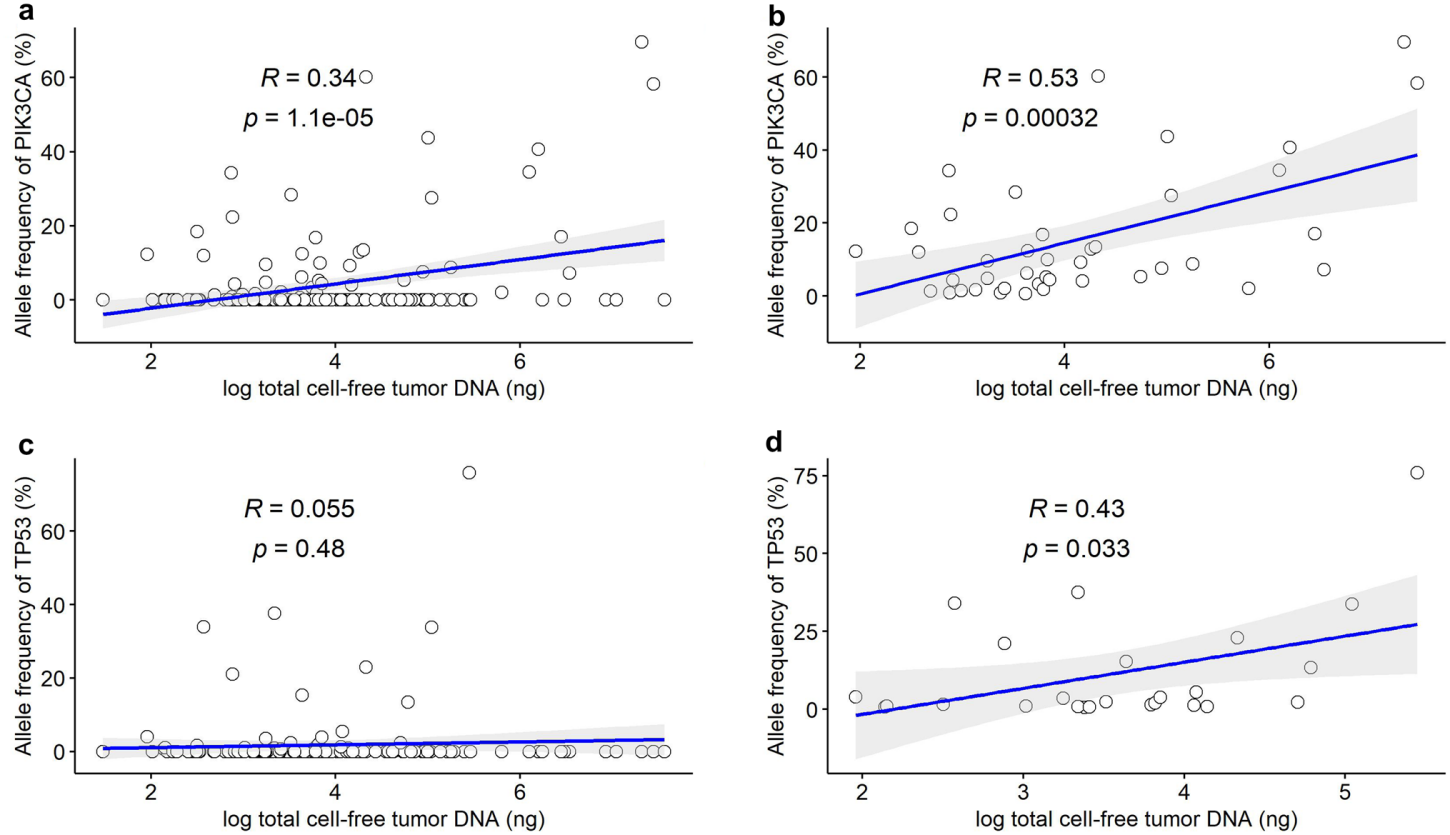
The correlation scatterplot of total ctDNA vs **a** PIK3CA (all patients), **b** PIK3CA (only patients with positive AF), **c** TP53 (all patients), **d** TP53 (only patients with positive AF). The axis of total ctDNA is in log10 scale. *AF* allele frequency. *ctDNA* cell-free tumor DNA

Because liquid biopsy by NGS is able to provide the allele frequency (AF) of each mutated variant, we aimed to understand if the AF of *PIK3CA* and *TP53* oncogenic mutations could be representative of total tumor burden. Thus, the correlation between total cfDNA (in ng) and AF (in %) of either *PIK3CA* or *TP53* mutation variants were examined. Patients with wild type *PIK3CA* or *TP53* were considered to have 0% AF. Inclusive of all patients, regardless of the status of the oncogenic mutations, the correlation between total cfDNA and *PIK3CA* and *TP53* AF was weak (Pearson correlation coefficient *R* = 0.34, *p* < 0.001) and non-existent (*R* = 0.055, *p* = 0.48), respectively (Fig. 3a, c). If the correlation analysis included only patients with *PIK3CA* or *TP53* mutations (AF > 0) identified by liquid biopsy, the correlation coefficient increased to 0.53 (*p* < 0.001, Fig. 3b) and 0.43 (*p* = 0.033, Fig. 3d) for *PIK3CA* and *TP53*, respectively. When the correlation analysis was limited to patients with hotspot *PIK3CA* mutations (H1047R, E545K, E542K, and H1047L, comprising 77% of PIK3CA mutations in our cohort, Fig. 1a), PIK3CA AF and cfDNA remained positively correlated (*R* = 0.38, *p* = 0.018). Thus, *PIK3CA* mutation AF in the liquid biopsy had a stronger correlation with tumor burden.

### Separating the effects of tumor burden and oncogenic mutations

**Fig. 4:**
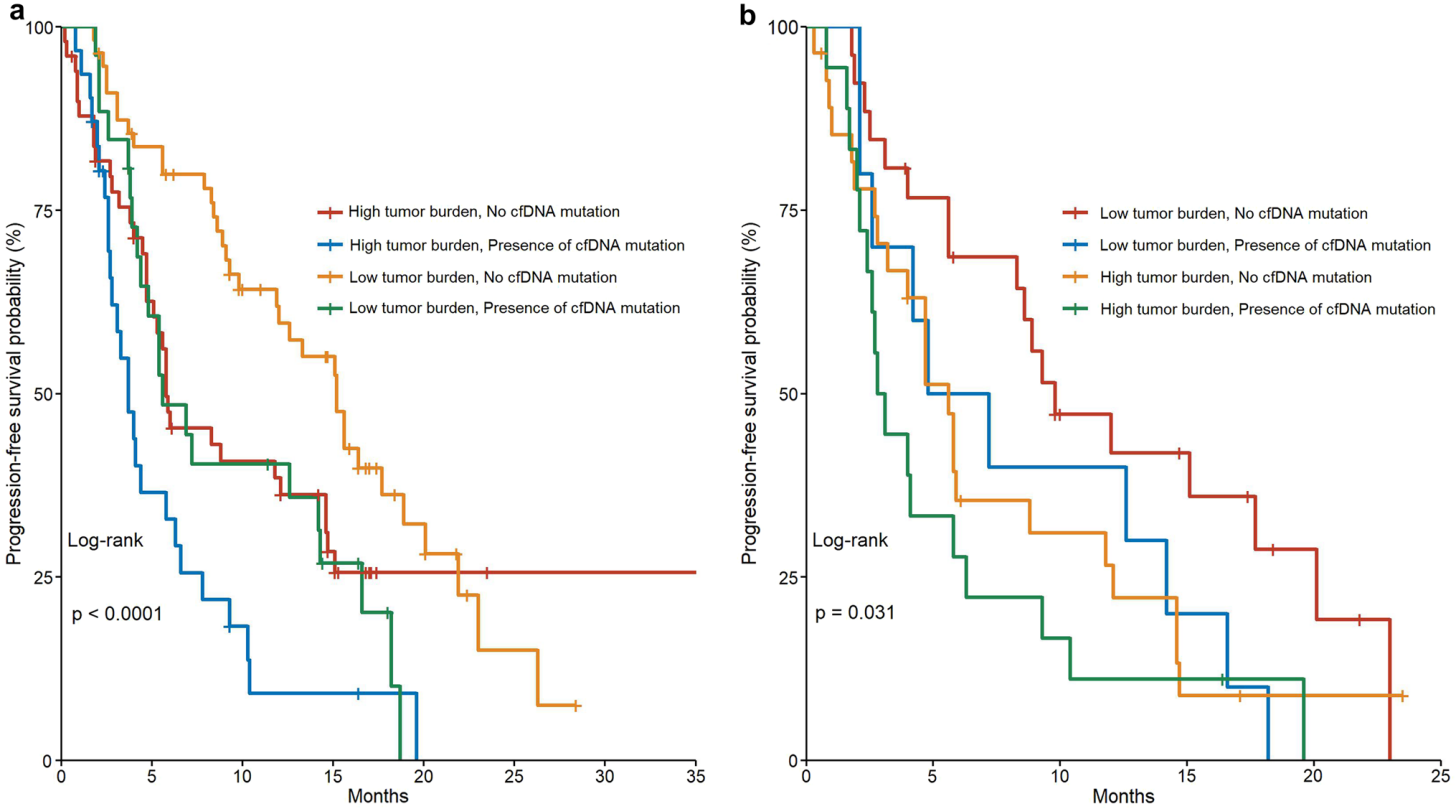
The Kaplan–Meier progression-free survival curves of ctDNA × oncogenic mutations **a** in the whole study cohort, **b** in patients treated with endocrine therapy plus everolimus

Based on the mutation status of *PIK3CA* and *TP53* mutation and the tumor burden, and we separated the patients into four groups (high vs low tumor burden and presence or no presence of cfDNA mutation). This was intended to understand the differential effect of these two factors on the treatment outcome of ET-based regimens. Not unexpectedly, patients with higher tumor burden and a presence of either *PIK3CA* or *TP53* oncogenic mutation had the worse outcome (*p* < 0.001) (Fig. 4a). Interestingly, patients with a presence of *PIK3CA* or *TP53* mutation but low tumor burden had similar PFS outcome with those with a high tumor burden but with no *PIK3CA* or *TP53* oncogenic mutations (median PFS 5.8 vs 5.6 months), suggesting that the oncogenic mutations of *PIK3CA* and/or *TP53* exerted additional resistance to the treatment of ET-base regimens in addition to the tumor burden status that was associated with worse PFS. These detrimental impact of *PIK3CA* or *TP53* on PFS were confirmed after adjusting for the lines of treatment and the type of treatment (HR 1.89 95% CI 1.29–2.79, *p* = 0.001). The HR for patients with either a PIK3CA or TP53 mutation receiving ET-based regimen as first, second, third and fourth line were 2.09, 2.15, 2.45, and 1.13, respectively. The p-value for interaction was 0.54.

In the preplanned subgroup analysis of patients received ET + everolimus (*n* = 78), the detrimental effect of high tumor burden (HR 1.70, 95% CI 1.04–2.78, *p* = 0.03) and *PIK3CA* and/or *TP53* oncogenic mutation (HR 1.67, 95% CI 1.02–2.76, *p* = 0.04) remained significant (Fig. 4b).

## Discussion

In addition to the less-invasive nature, liquid biopsies contain genetic alterations that reflect intra- and inter-tumoral heterogeneity, which is valuable for direct personalized therapies. In our prospective study of 163 patients, we showed that total cfDNA and the presence of either *PIK3CA* or *TP53* oncogenic mutation from liquid biopsy before the start of endocrine-based therapy were associated with worse PFS in ER + /HER2- MBC patients. In addition, after adjustment of other clinical variables such as the lines and the type of treatment, these two factors remained significantly associated with PFS. The prospective nature of the study and that we limited the recruitment to patients treated with ET-based regimens in a more homogenous population further lends strength to our findings.

While studies have reported the association between a worse outcome and the presence of *PIK3CA* or *TP53* mutation, few included the tumor burden as a variable to adjust the impact of these oncogenic mutations. The tumor burden of ER-positive MBC may be especially hard to estimate because of the propensity for bone metastases. Although other factors such as peripheral blood mononuclear cell lysis or normal tissue shedding that could confound the interpretation of cfDNA, we specifically selected shorter fragments of cell-free DNA that are associated with tumor DNA [18]. Our findings that the amount of total cfDNA were inversely associated with PFS supports that total cfDNA may be considered as a surrogate marker of tumor burden in ER-positive MBC patients. However, we need to be cautious to use total cfDNA solely as a surrogate marker to assess tumor burden, which, while important, is complicated and has no standard procedures based on liquid biopsy results at present. Factors that could influence the interpretation of cfDNA such as tumor DNA shedding and the mutation fraction should also be considered before implantation of cfDNA as the sole biomarker for tumor burden.

Few prospective studies have accounted for the tumor burden when assessing the impact of *PIK3CA* and *TP53* mutations on ET-based treatment based on liquid biopsy results. In this study, the inverse association of *PIK3CA*/*TP53* mutations with PFS was evident in patients with high and low tumor burdens. Previous studies showed that *PIK3CA* mutation rate did not differ significantly between primary tumors and corresponding metastases, but the prognostic effect was stage-dependent. In early stage breast cancer patients, the presence of *PIK3CA* mutation was associated with a better disease-free survival and overall survival [21]. But in ER-positive metastatic breast cancer, the presence of *PIK3CA* mutation exerted a negative effect on survival [11]. The transition from favorable to the inferior impact of *PIK3CA* mutations supported our findings that *PIK3CA* had a significant negative survival impact at the early stage of metastatic disease and conferred resistance to treatments.

The findings that *PIK3CA* mutation in the cfDNA is a prognostic marker for worse PFS in ET-based regimens have multiple clinical implications. A recent randomized phase III study, SOLAR-1, demonstrated the superior efficacy of the combination of fulvestrant and alpelisib, a PI3k-alpha specific inhibitor, as compared to fulvestrant alone in ER-positive, HER2-negaitve MBC [22]. The benefit of alpelisib may even be stronger in patients whose *PIK3CA* were detected by cfDNA [23]. However, the benefit of alpelisib seemed to be more modest when used in a later line setting [24]. Incorporating our findings that PIK3CA mutation portends a worse prognosis even when tumor burden is low, patients should consider specific *PIK3CA* inhibitor in the earliest time after failure of standard first-line treatment of ET plus a CDK4/6 inhibitor.

Liquid biopsy may provide the variant AF of multiple genetic mutations in a single test but debate remains as to the variant AF of which gene may best represent tumor burden. In our study, the two genes selected for NGS are both common mutations of breast cancer. However, *PIK3CA* is an oncogene where hotspots are mostly single nucleotide variant and *TP53* is a tumor suppressor gene that various types of genetic alterations could lead to a dysfunctional p53 protein [15, 25]. Our results suggested that in patients with *PIK3CA* mutation identified by liquid biopsy, the variant AF of *PIK3CA* strongly correlated with tumor burden. However, the correlation between *TP53* variant AF and total cfDNA was less pronounced and the reason may be that amplicon-based NGS is unable to detect larger deletions, loss of heterozygosity, and structural variations. Thus, when an amplicon-based NGS panel is used for liquid biopsy testing, the variant AF of a oncogene mutation may provide a better reflection of tumor burden than tumor suppressor genes. However, the correlation between TP53 variant AF and total cfDNA was less pronounced, and the reason may be that amplicon-based NGS is unable to detect larger deletions, loss of heterozygosity, and structural variations. Thus, when an amplicon-based NGS panel is used for liquid biopsy testing, based on our study results, we propose that the variant AF of a bona fide oncogene mutation may better reflect tumor burden than tumor suppressor genes. Newer NGS technology that could detect a wider range of genomic alterations and structural variations associated with tumor suppressor genes in liquid biopsy could be more precise in using AF to reflect tumor suppressor gene mutated clones. Investigators could further validate this hypothesis and determine how to optimally use AF of various gene alterations in this era that dozens of genetic alterations are within reach in liquid biopsy samples. Furthermore, the dynamic changes of the AF of ctDNA in liquid biopsy could also reflect the changes of tumor heterogeneity (intra- and inter-tumor) and may reveal the potential resistance mechanism to ET-based regimens.

The success of BOLERO-2 study confirmed the role of everolimus in the treatment of ER-positive HER2-negative MBC [26]. Retrospective analysis of archival tumor specimens and droplet-digital PCR-based liquid biopsy from BOLERO-2 showed that the presence of *PIK3CA* mutation was a prognostic but not predictive biomarker [12, 27]. Although *TP53* mutation status was examined, there was no formal report regarding the effect of *TP53* on the treatment efficacy of exemestane plus everolimus in BOLERO-2 [12, 27]. Through NGS-based liquid biopsy, we prospectively confirmed that *PIK3CA* and *TP53* mutations conferred a detrimental effect with shorter PFS in patients treated with the combination of ET + everolimus.

Our study has some caveats. It is widely accepted that the combination of an ET plus CDK4/6 inhibitor is the first-line treatment standard for ER-positive/HER2-negative MBC, but only 14% of the study population received first-line CDK4/6 inhibitors. Nested studies from phase III CDK4/6 inhibitor clinical trials have suggested that the presence of PIK3CA mutation did not have significant detrimental impact on PFS. [13, 28]. Thus, our conclusion that *PIK3CA* mutation is a marker for worse outcome for ET-based regimens in ER-positive MBC should be cautiously interpreted in the first-line setting with ET and CDK4/6 inhibitor combinations. Secondly, our analysis did not cover the entire coding regions of genes selected, which may account for the lower than expected mutation rate of *TP53*. In addition, large-segment deletions or gene rearrangements analysis is not allowed due to the limited length of cfDNA. However, because more than 95% of *PIK3CA* mutations in BC are hotspot mutations [29, 30], we are confident that our study design could pick up most of the *PIK3CA* mutations. Lastly, the amount of cfDNA could be influenced by the total amount of plasma extracted from patients. Although we did not record each sample’s exact whole blood and subsequent plasma volume, the protocol-based standardized procedures of blood draw, plasma separation, and cfDNA extraction made us believe that the error (bias) would more likely to be randomly distributed and have limited impact to the results.

In conclusion, we demonstrated plasma *PIK3CA* and *TP53* mutations are independent predictive markers for shortened PFS in ER-positive BC receiving ET-based regimens. Besides, the level of plasma cfDNA and *PIK3CA*/*TP53* AF could be a surrogate for tumor burden to correlate treatment outcomes. Liquid biopsy in ER-positive/HER2-negative MBC patients is achievable, provides prognosis information, and may also lead to different selection of treatment of the patients.

## Supplementary Information

Below is the link to the electronic supplementary material.

Supplementary file1 (TIFF 7607 KB)—**Fig. 1** The distribution of extracted cell-free DNA (cfDNA). The dashed line indicates the median

Supplementary file2 (JPG 1557 KB)—**Fig. 2** The Kaplan-Meier curve of median progression-free survival (PFS) of patients based on the mutation status of PIK3CA and TP53. *MT* mutation, *WT* wild type

## Data Availability

The datasets generated and analysed during the current study are not publicly available becuase it was not permitted by our Research Ethics Committte. but may be available from the corresponding author on reasonable request.
