# Supplementary figures and images for: Plasma cell-free tumor DNA, PIK3CA and TP53 mutations predicted inferior endocrine-based treatment outcome in endocrine receptor-positive metastatic breast cancer
Source: Breast Cancer Res Treat. 2023 Jun 21;201(3):377–85. doi: 10.1007/s10549-023-06967-3 (PMC10460702; doi:10.1007/s10549-023-06967-3)

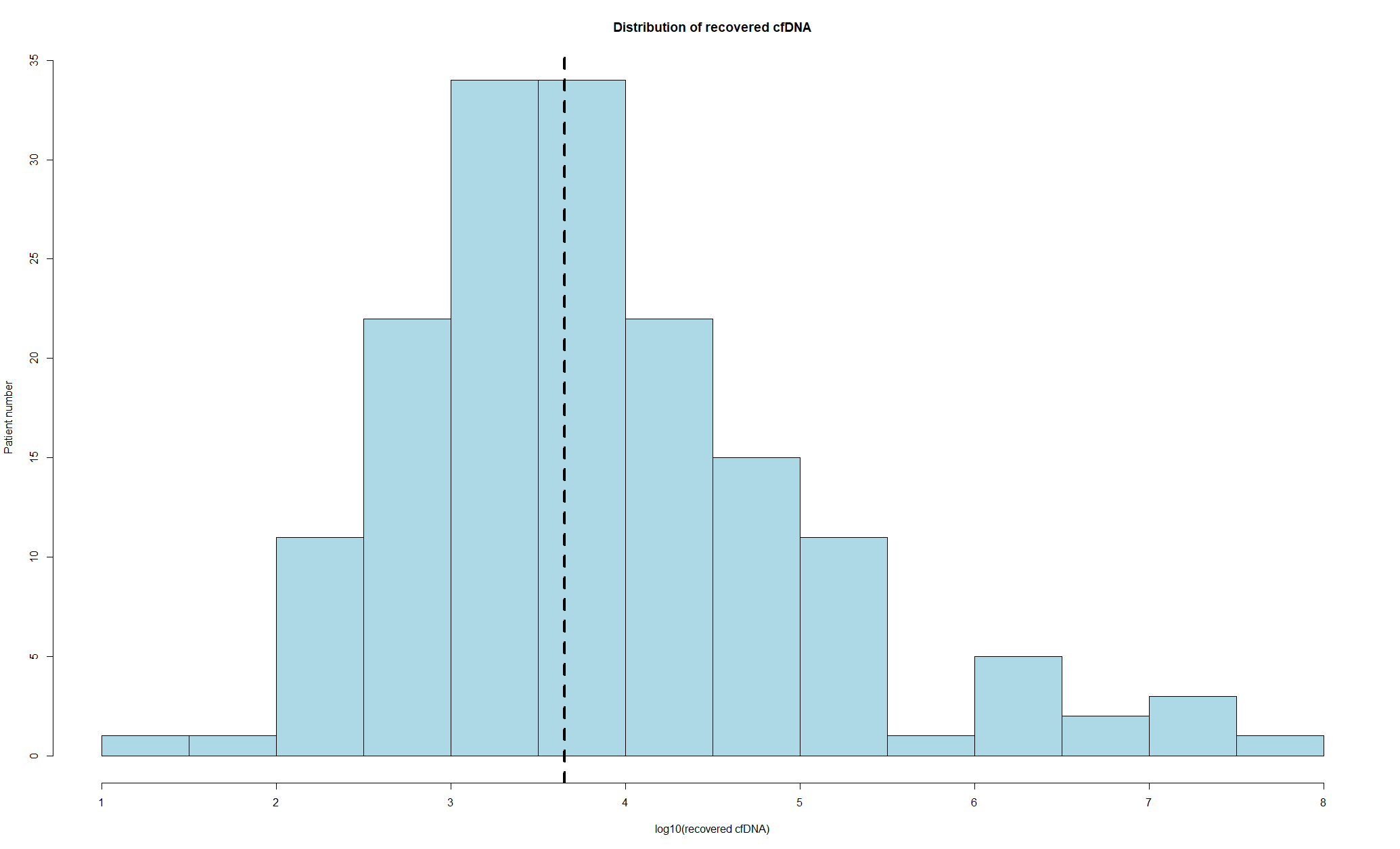

Supplement: Supplementary file 1 — Supplementary file1 (TIFF 7607 KB)—Fig. 1 The distribution of extracted cell-free DNA (cfDNA). The dashed line indicates the median [file 10549_2023_6967_MOESM1_ESM.tiff]

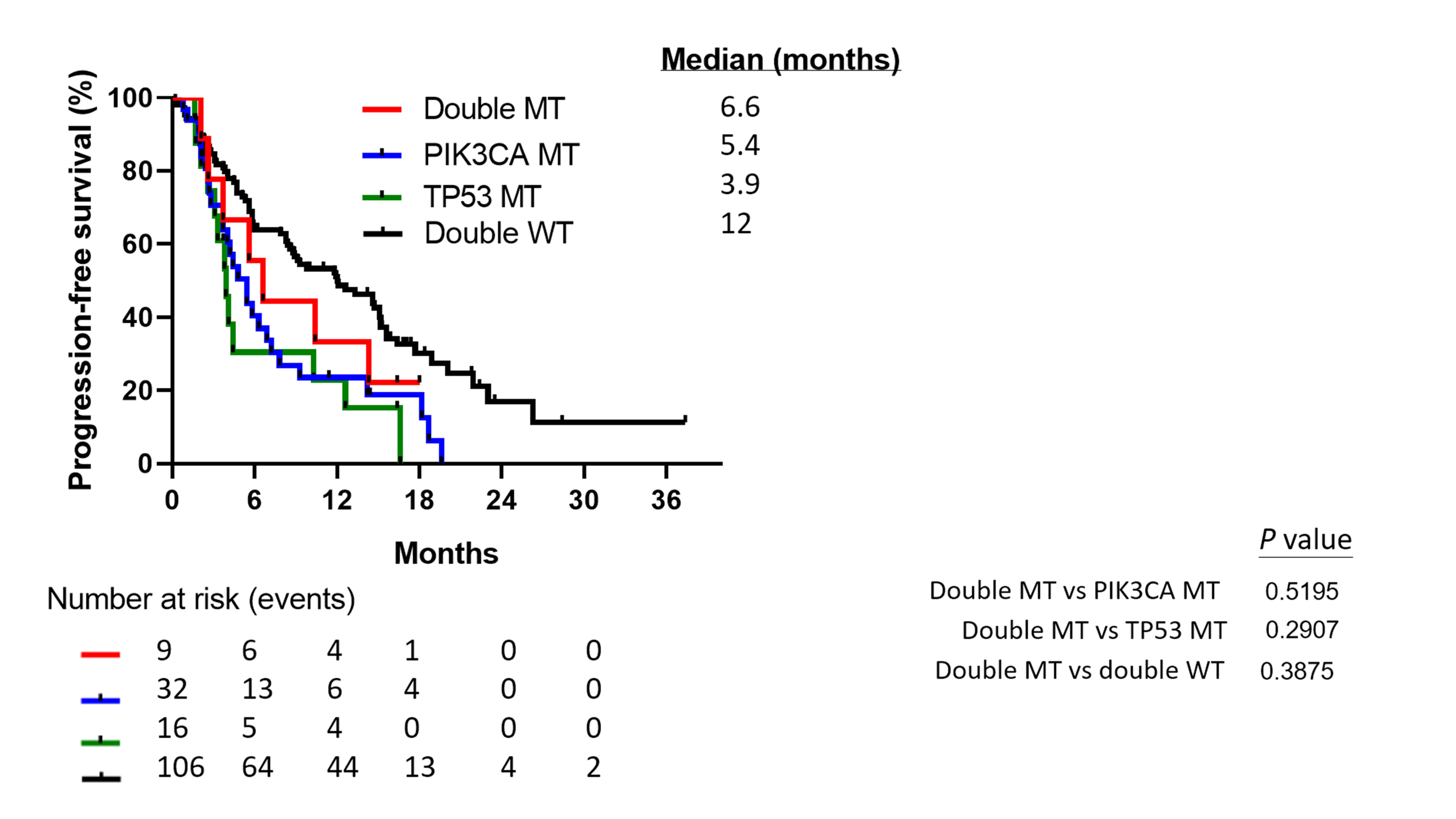

Supplement: Supplementary file 2 — Supplementary file2 (JPG 1557 KB)—Fig. 2 The Kaplan-Meier curve of median progression-free survival (PFS) of patients based on the mutation status of PIK3CA and TP53. MT mutation, WT wild type [file 10549_2023_6967_MOESM2_ESM.jpg]
